# The Effects of CoCl_2_ on HIF-1α Protein under Experimental Conditions of Autoprogressive Hypoxia Using Mouse Models

**DOI:** 10.3390/ijms150610999

**Published:** 2014-06-18

**Authors:** Yan-Bo Zhang, Xiulian Wang, Edward A. Meister, Ke-Rui Gong, Shao-Chun Yan, Guo-Wei Lu, Xun-Ming Ji, Guo Shao

**Affiliations:** 1 Department of Neurology, Affiliated Hospital of Tai Shan Medical University, Taishan 271000, China; E-Mail: bbnnbn@163.com; 2 Department of Intensive Care Unit , 2nd Affiliated Hospital of Baotou Medical College, Baotou 014030, China; E-Mail: zhangjianjunnmg@163.com; 3 Department of Medicine, University of Arizona, Tucson, AZ 85721, USA; E-Mail: eemeister@yahoo.com; 4 Biomedicine Research Center and Basic Medical College, Baotou Medical College, Baotou 014060, China; E-Mails: keruigong@gmail.com (K.-R.G.); yanshaochunby@163.com (S.-C.Y.); 5 Department of Anatomy, University of California, San Francisco, CA 94143, USA; 6 Institute for Hypoxia Medicine, Xuanwu Hospital of Capital Medical University, Beijing 10054, China; E-Mail: gwlu32@163.com

**Keywords:** hypoxia preconditioning, hypoxia-inducible factor-1, hippocampus, CoCl_2_

## Abstract

It is well known that cobalt chloride (CoCl_2_) can enhance the stability of hypoxia-inducible factor (HIF)-1α. The aim of this study is to detect the effect of CoCl_2_ on the hypoxia tolerance of mice which were repeatedly exposed to autoprogressive hypoxia. Balb/c mice were randomly divided into groups of chemical pretreatment and normal saline (NS), respectively injected with CoCl_2_ and NS 3 h before exposure to hypoxia for 0 run (H0), 1 run (H1), and 4 runs (H4). Western Blot, electrophoretic mobility shift assay (EMSA), extracellular recordings population spikes in area *cornus ammonis* *I* (CA 1) of mouse hippocampal slices and real-time were used in this study. Our results demonstrated that the tolerance of mice to hypoxia, the changes of HIF-1α protein level and HIF-1 DNA binding activity in mice hippocampus, the mRNA level of erythropoietin (*EPO*) and vascular endothelial growth factor (*VEGF*), and the disappearance time of population spikes of hippocampal slices were substantially different between the control group and the CoCl_2_ group. Over-induction of HIF-1α by pretreatment with CoCl_2_ before hypoxia did not increase the hypoxia tolerance.

## 1. Introduction

It has been demonstrated that a sublethal ischemic/hypoxic exposure can improve the tolerance of tissue and/or cells to a subsequent lethal ischemic/hypoxic insult. This phenomenon is called ischemic/hypoxic preconditioning (I/HPC) and was first observed in the dog heart and later in the gerbil brain [1,2]. Modification of the tolerance of neurons to hypoxia was demonstrated using various chemicals [3,4]. Hence, endogenous cellular adaptation or suitable exogenous chemical stimulation will increase the hypoxia tolerance of a cell or an organ. We developed a unique animal model of repetitive autohypoxia for whole body I/HPC (WBHPC) in adult mice [5,6]. The salient aspect of this animal model is that the tolerance to hypoxia is increased with each exposure, and functions in an essentially linear arithmetic progression [7]. Using this model we have observed changes in molecules of the brain, including hypoxia-inducible factor (HIF)-1α, in developing hypoxic preconditioning in autoprogressive hypoxia [7,8,9]. We propose that HIF-1α can play an important role in contributing to neuron protection [7].

HIF-1α (located on Chromosome 14 q21–q24) is a subunit of the HIF-1 which is transcription factor responsive to alterations in cellular oxygen levels. HIF-1α is a target of prolyl hydroxylation by HIF enzyme prolyl-hydroxylase. Under normoxic conditions HIF-1α is synthesized and subjected to rapid hydroxylation on two proline residues; 564 and 402. When conditions are hypoxic HIF prolyl hydroxylase is inhibited because it uses oxygen as a co-substrate [10]. This hydroxylase inhibition increases HIF-1α stability and as such impairs the binding of von Hippel-Lindau protein (pVHL) to HIF-1α, resulting in steady accumulation of HIF complexes within the cell nucleus [11]. Hydroxylase inactivity allows HIF-1α to become transcriptionally active for the up-regulation of a large number of hypoxia-sensitive genes [12]. HIF-1α binds to the core DNA sequence of 5'-[AG]CGTG-3' at the hypoxia response element of target gene promoters. Hydroxylase can regulate the stability of HIF-1α, while other factors can affect its degradation. Liu and colleagues have demonstrated that the Receptor for Activated C Kinase1 (RACK1) protein can bind to HIF-1α and subsequently, by recruiting an E3 ubiquitin-protein ligase complex, can replace von Hippel-Lindau protein to initiate degradation of HIF-1α. C-jun is associated with HIF-1α via its oxygen-dependent degradation domain, masks the sites for ubiquitination and thus protects HIF-1α from proteasome-executing degradation [13].

It has been reported that cobalt, a transition metal, directly inhibits the interaction between pVHL and hydroxylated HIF-1 causing the stabilization of HIF-1α [14,15]. Thus, HIF-1α escapes degradation and subsequently dimerizes with HIF-1β leading to the active HIF-1 transcription factor (TF) binding to the hypoxic response element of the promoter region of many target genes. This process leads to the transcriptional activation of several dozen genes that may protect the neuron cells from hypoxic damage [16,17,18]. Hence, cobalt can be used to mimic hypoxia to increase HIF-α [17,19]. We have proposed that HIF-1α can play a center role in the tolerance time in autoprogressive hypoxic mice [20]. However, Vangeison *et al.* reported that loss of HIF-1α function in neurons reduced neuronal viability during hypoxia. This selective loss of HIF-1 function in astrocytes markedly protected neurons from hypoxia-induced neuronal death [21]. In order to better understand the underlying mechanisms involved, we measured the change in hypoxic tolerance in mice that were exposed to repetitive auto-hypoxia after HIF-α induced by cobalt pretreatment.

## 2. Results and Discussion

### 2.1. The Effect of CoCl_2_ Pretreatment on Hypoxia Tolerance

**Figure 1:**
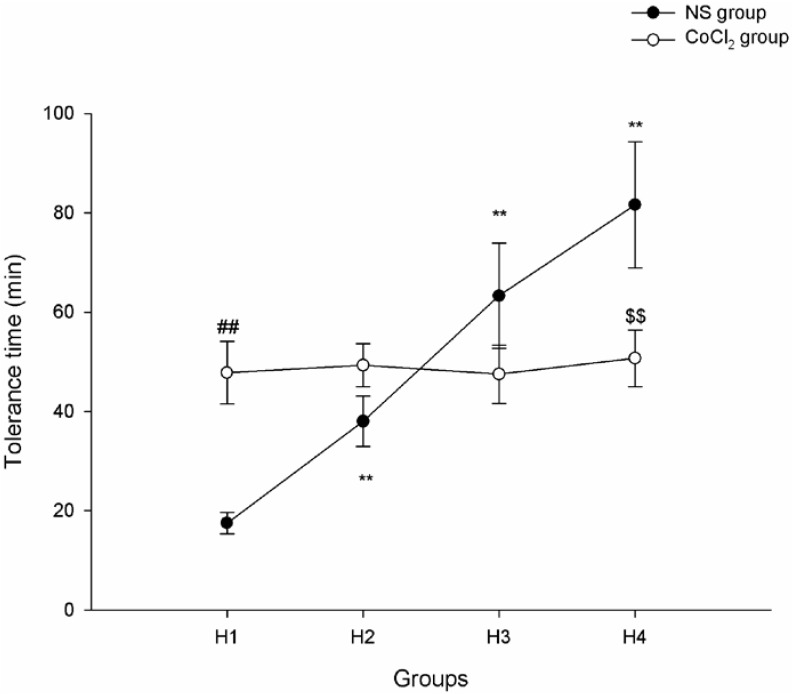
Tolerance time of mice exposed to hypoxia for 1–4 runs. (*n* = 10; ** *p* < 0.01 as compared with the preceding runs in normal saline (NS) group; ## *p* < 0.01 H1_NS_ *vs.* H1_CoCl2_; $$ *p* < 0.01 H4_NS_ *vs.* H4_CoCl2_).

In the normal saline (NS) group, the tolerance time of each runs lasted significantly longer and longer, as the exposure run increased. The average hypoxic tolerance times of runs 1, 2, 3 and 4 (showed as H1, H2, H3 and H4) were 17.5 ± 2.2, 38.0 ± 5.1, 63.3 ± 10.6 and 81.6 ± 12.7 min, respectively (*p*-value < 0.01). In the CoCl_2_ group_,_ the average tolerance times of runs 1, 2, 3 and 4 were 47.8 ± 6.3, 49.3 ± 4.3, 47.5 ± 5.9 and 50.7 ± 5.7 min, respectively (*p*-value > 0.05). The tolerance time of H1 mice in the NS group was lower than that of H1 mice in the CoCl_2_ group (*p*-value < 0.01). On the contrary, the tolerance time of H4 mice in the NS group was higher than that of H4 mice in the CoCl_2_ group (*p*-value < 0.01) (Figure 1).

### 2.2. The Effect of CoCl_2_ Pretreatment on Hypoxia-Inducible Factor (HIF)-1α Protein Level

**Figure 2:**
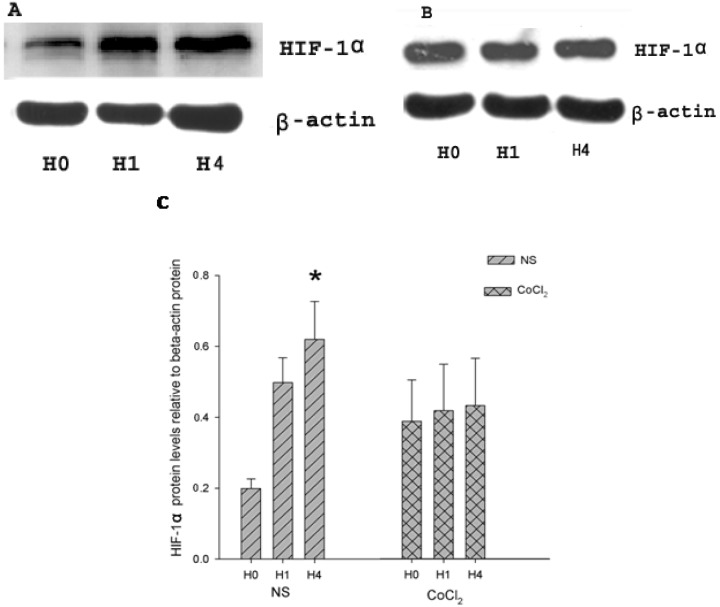
Expression of hypoxia-inducible factor (HIF)-1α and β-actin in hippocampus. (**A**) Western blot analysis of HIF-1α and β-actin content in the hippocampus of hypoxic preconditioned mice pretreated with NS; (**B**) Western blot analysis of HIF-1α and β-actin content in the hippocampus of hypoxic preconditioned mice pretreated with CoCl_2_; (**C**) Relative abundance of HIF-1á protein in hippocampus of hypoxia-preconditioned mouse treated with CoCl_2_ and NS, respectively. (*n* = 4; * *p* < 0.05 * vs.* H0 and H1 in NS group).

HIF-1α protein and β-actin (internal control) protein were detected in CoCl_2_ and NS groups by Western Blot (Figure 2A,B), using a goat polyclonal antibody (Santa Cruz Biotechnology, Santa Cruz, CA, USA) and a mouse monoclonal antibody (Sigma Aldrich, St. Louis, MO, USA). HIF-1α protein levels were presented by relative abundance (RA, optical density (OD) of HIF-1α protein/OD of β-actin protein). In the NS group, a faint band of about 120 kDa was observed in H0 (RA 0.199 ± 0.027), while a more distinct band of HIF-1α protein appeared at the corresponding location in H1 and H4. The HIF-1α protein level was increased in both group H1 (RA 0.498 ± 0.07) and group H4 (RA 0.620 ± 0.107). The HIF-1α protein levels in group H4 were significantly higher than those in group H0 and H1 (*p* < 0.01 *vs.* H0 and H1, *n* = 4; Figure 2C). In CoCl_2_ group, the HIF-1α protein band can be clearly anchored in H0 (RA 0.387 ± 0.117), HI (RA 0.417 ± 0.131) and H4 (RA 0.432 ± 0.133). No difference emerged among the three groups in CoCl_2_ treatment mice (*p*-value > 0.05; Figure 2C).

### 2.3. The Effect of CoCl_2_ Pretreatment on HIF-1 DNA Binding Activity

**Figure 3:**
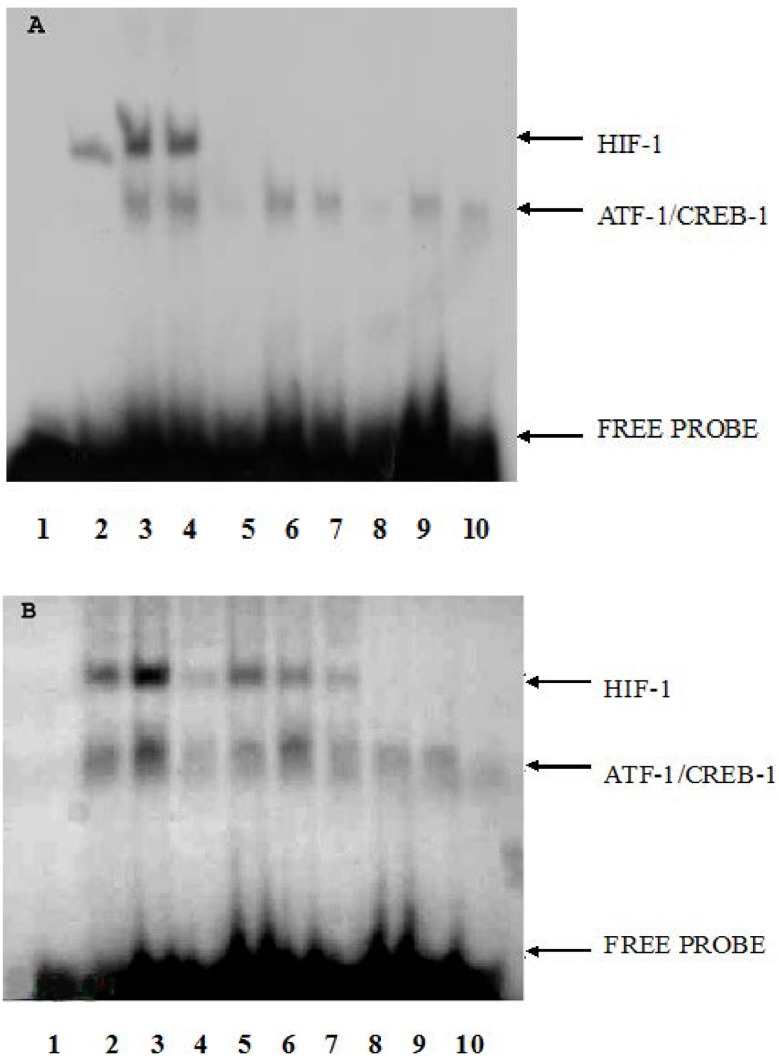
DNA-binding activity of HIF-1 on the EPO hypoxia response element (HRE) in hippocampus of mice in different groups. Lane 1: protein free. EMSA using crude nuclear extract was prepared from H0 (lanes 2, 5, 8), H1 (lanes 3, 6, 9), and H4 (lanes 4, 7, 10). For supershift assays (lanes 5–7), binding reactions contained 2ìl antiserum raised against HIF-1α. For competition assays (lanes 8–10), binding reactions included 100-fold excess of unlabeled oligonucleotides. (**A**) NS group; (**B**) CoCl_2_ group (*n* = 3).

**Figure 4:**
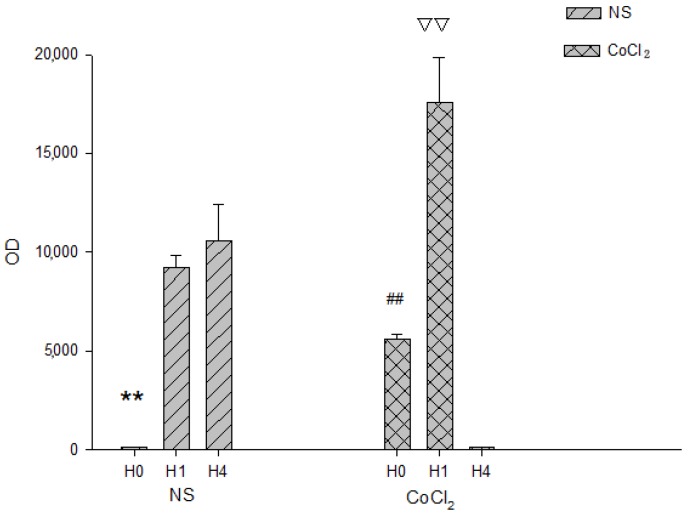
The HIF-1 DNA binding activity in hippocampus of hypoxia-preconditioned mouse treated with CoCl_2_ and NS, respectively. (*n* = 3; ** *p* < 0.01 *vs.* H1 and H4 in NS group; ## *p* < 0.01 *vs.* H1 and H4 in CoCl_2_ group; ▽▽ *p* < 0.01 *vs.* H0 and H4 in CoCl_2_ group).

The DNA-binding activities in the NS and CoCl_2_ groups were analyzed by electrophoretic mobility shift assay (EMSA) (Figure 3). The supershift assays and competition assays were used to determine the specificity of erythropoietin (EPO) hypoxia response element (EPO HRE)/HIF-1 interaction as well as to identify the HIF-1 involved in complex formation. In the NS group, the DNA-binding activities of HIF-1 was very low in H0 (OD 168 ± 11), and increased in H1 (9210 ± 630) and greatly increased in H4 (OD 10,602 ± 1791). A significant difference in the OD value of HIF-1 DNA-binding activities was observed among H0, H1 and H4 in NS group (*p*-value < 0.01, *n* = 3; Figure 4). In the CoCl_2_ group, the DNA-binding activities of HIF-1 in H0 were lower (OD 5602 ± 238) than those in H1 (OD 17,591 ± 2230) and higher than those in H4 (OD 129 ± 25). The HIF-1 DNA-binding activities increased in H1 while they decreased in H4 (*p*-value < 0.01, *n* = 3; Figure 4). DNA-binding activities of each group were calculated by optical density (OD) value of bands on X-ray film. Because OD values of EMSA bands depend on the development time of X-ray film and the OD values of CoCl_2_ group and NS group were obtained from different X-ray film, the OD values between the CoCl_2_ group and NS group were not compared.

### 2.4. The Effect of CoCl_2_ Pretreatment on the Erythropoietin (EPO) and Vascular Endothelial Growth Factor (VEGF) mRNA Levels

**Figure 5:**
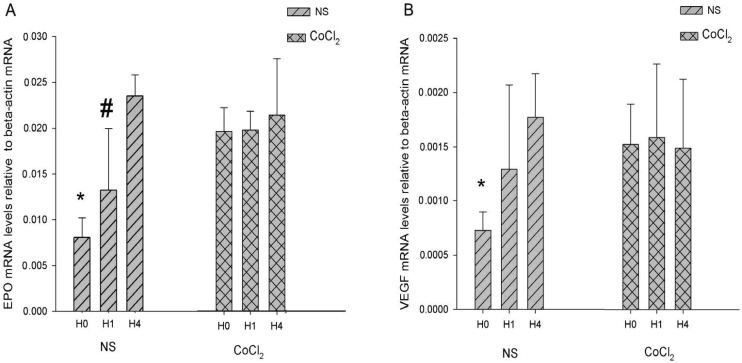
Real-time PCR analysis *erythropoietin* (*EPO*) and *vascular endothelial growth factor* (*VEGF*) mRNA in hippocampus. (**A**) The *EPO* mRNA level relative to β-*actin* mRNA; (**B**) the *VEGF* mRNA level relative to β-actinmRNA. (*n* = 6; * *p* < 0.05 *vs.* H1 and H4 in NS group; # *p* < 0.05 *vs.* H0 and H4 in NS group).

The two target genes, *EPO* and *vascular endothelial growth factor* (*VEGF*), mRNA of HIF-1 were analyzed by real-time PCR. Relative *EPO* and *VEGF* mRNA levels were standardized by β-*actin* mRNA. The changes of them were very similar. In NS group, the *EPO* and *VEGF* mRNA levels were increased in H1 and H4 respectively. A significant difference in the relative abundance of *EPO* mRNA to β-*actin* mRNA was observed among H0 (0.0081 ± 0.0021), H1 (0.0132 ± 0.0063) and H4 (0.0235 ± 0.0027) in NS group (*p*-value < 0.05, *n* = 6; Figure 5A), while the relative abundance of *VEGF* mRNA in H0 (0.0007 ± 0.0002) was found lower than that in H1 (0.0012 ± 0.0007) and H4 (0.0017 ± 0.0004) (*p*-value < 0.05, *n* = 6; Figure 5B). In CoCl_2_ group, no differences were discerned in *EPO* mRNA among the H0 (0.0163 ± 0.0021), H1 (0.0164 ± 0.0017), H4 (0.0178 ± 0.0052) and in *VEGF* mRNA among the H0 (0.0015 ± 0.0003), H1 (0.0016 ± 0.0006) and H4 (0.0015 ± 0.0006) (*p*-value > 0.05, *n* = 6; Figure 5). The change of *EPO* and *VEGF* mRNA levels agreed with HIF-1 binding activity in the NS group and did not agree with HIF-1 binding activity in the CoCl_2_ group. We found that *EPO* and *VEGF* mRNA levels were higher at H0 and lower at H4 in the CoCl_2_ group compared with those in NS group. These results may imply an increase of *EPO* and *VEGF* mRNA in the NS group during hypoxia exposure while their expression is already higher in the CoCl_2_ group at H0 and no longer increases during the H1–H4 periods.

### 2.5. The Effect of CoCl_2_ Pretreatment on the Abolishment Time of Population Spikes (PS)

**Figure 6:**
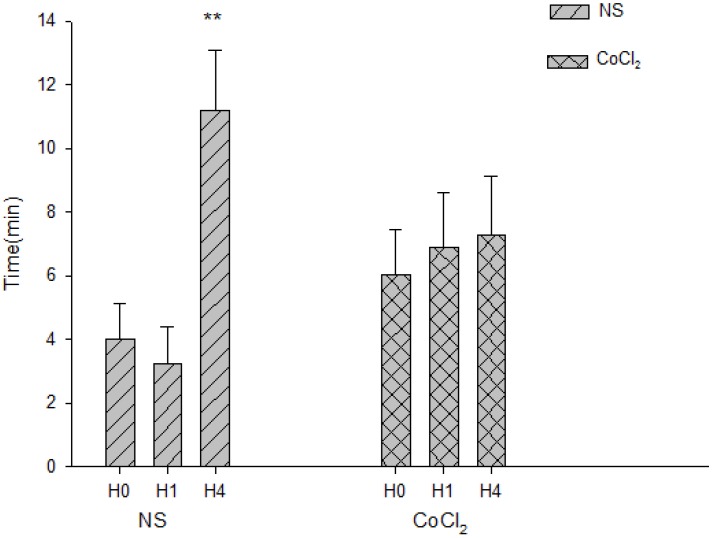
The abolishment time of population spikes(PS) in hippocampal slices of hypoxia-preconditioned mice treated with CoCl_2_ and NS, respectively. (*n* = 9; ** *p* < 0.01, *vs.* H0 and H1 in NS group).

Extracellular recordings of electrically-evoked population spikes (PS) in the stratum pyramidale of the CA1 region were used to test the synaptic function. The amplitude of PS was decreased and even disappeared implying the decrease or loss of synaptic function under hypoxic conditions. The abolishment time of PS can be used to measure the change of hypoxic resistance to hypoxia simulation [22]. In the NS group, the abolishment time of PS during H0, H1 and H4 was 4.01 ± 1.11, 3.25 ± 1.17 and 11.18 ± 1.92 min, respectively. The abolishment time of H4 was longer than that of H0 and H1 (*p*-value < 0.01, *n* = 9, Figure 6D) in the NS group. In the CoCl_2_ group, the abolishment time of PS of H0, H1 and H4 was 6.02 ± 1.42, 6.88 ± 1.73 and 7.27 ± 1.87 min respectively. There were no statistically significant differences among H0, H1 and H4 in the CoCl_2_ group (*p*-value > 0.05, *n* = 9, Figure 6).

The present study was undertaken to observe the effects of CoCl_2_ pretreatment on hypoxic tolerance using an autoprogressive hypoxia mice model. Because many studies have shown that the CoCl_2_ preconditioning can reduce the ischemia/hypoxia injury in many fields [23,24], our study focused on tolerance time and the disappearance of PS in hypoxia preconditioning mice which were treated with CoCl_2_ before hypoxia exposure. Through this study we further clarified the role of HIF-1 in hypoxia tolerance.

Hypoxia and CoCl_2_ can prevent the pVHL tumor suppressor protein, an ubiquitin protein ligase, from binding HIF-1α, by which HIF-1α escapes the degradation caused by the ubiquitin pathway under hypoxic conditions or CoCl_2_ treatment [14]. In the NS group, the level of HIF-1α increased in H1 and remarkably increased in H4. Interestingly no difference was discerned in HIF-1α protein level in CoCl_2_ treatment mice that were exposed to hypoxia once, four times, or not at all. Similarly to our results, Shrivastava *et al.* showed no significant differences in the HIF-1 protein level among the hypoxia group, CoCl_2_ group, or the CoCl_2_ + hypoxia group [17]. The reason why hypoxia combined with CoCl_2_ treatment did not increase HIF-1α protein levels is unclear. We propose that CoCl_2_ may be a very efficient drug that can totally inhibit the activation of pVHL, and that HIF-1α may have reached its peak level after 3 h of CoCl_2_ treatment (Figures S1 and S2). Therefore, hypoxia and hypoxia preconditioning could not increase the HIF-1 protein level of the CoCl_2_ pretreatment animal. At the same time, we found that cobalt treatment significantly attenuated HIF-1 DNA binding activity. A similar report showed that a reduction of HIF-1 binding activity was also observed when neurons were preconditioned by a non-lethal oxygen-glucose deprivation (OGD) interval (60 min), 48 h prior to the 90 min OGD. Thus, hypoxic preconditioning reduced activation of HIF-1 binding activity [25]. We postulate that there must be some molecules or materials that attenuate the activity to prevent cell damage from long-term high activity in spite of the high protein level [26]; however, the mechanism of the decrease HIF-1 binding activity remains to be elucidated.

HIF-1 DNA binding activity may be the most important factor for hypoxic tolerance. Expression of HIF-1 downstream gene products EPO and VEGF have been found to protect neuronal cells from direct hypoxic/ischemic injury [27,28]. In our results, we posited that EPO and VEGF may play neuroprotective roles directly in NS and CoCl_2_ groups. Some HIF-1 downstream gene products may counteract detrimental hypoxic/ischemic injury indirectly. For example, Nucleophosmin (NPM) forced expression by HIF-1α can inhibit hypoxia-induced p53 phosphorylation at Ser-15 and interacts with p53, which suppresses p53 activation and maintains cell survival in hypoxic cells [29]. Similar to the change of HIF-1α in the NS and CoCl_2_ groups, our results showed that two of HIF-1 target gene, *EPO* and *VEGF*, were increased under hypoxic condition and were not increased under CoCl_2_ + hypoxia condition. We propose that expression of some HIF-1 target genes, such as *EPO* and *VEGF*, may reach a high level induced by CoCl_2_ pretreatment and are no longer induced by hypoxia. Jones *et al.* reported that newborn rats preconditioned with CoCl_2_ did not produce any change in glucose transport and glycolysis gene (HIF-1 target gene) expression in brain [30]. The molecular mechanisms of hypoxia tolerance may be that some HIF-1 target genes induced by CoCl_2_ pretreatment to high levels cannot be induced by hypoxia after CoCl_2_ pretreatment. This may explain why the tolerance time and the PS abolishment time of hippocampal slices were different between the NS and CoCl_2_ groups in our study.

Hippocampal slice preparations have been used extensively for many years to study various biochemical and electrophysiological alterations produced by oxygen deprivation in the mammalian central nervous system [31]. Hypoxia induced depression of electrical activity was associated with ATP insufficiency which leads to decreased membrane resistance due to an increase in potassium. The abolishment time of PS can be regarded as an indicator of synaptic transmission for hypoxic tolerance [22]. Forment *et al.* demonstrated that the HIF–VHL pathways have significant effects on human energy metabolism at the level of the organism as a whole [11]. Some of the HIF-1 target proteins, for example cytoglobin and VEGF [32,33,34], may increase oxygen supply for the energy demand in the brain to postpone the abolishment time of PS. Thus, the differences in abolishment time of PS between the CoCl_2_ and NS groups may be due to the changes of HIF-1 DNA binding activity and possibly some of the HIF-1 target genes in our study.

While HIF-1 DNA binding activity contributed to the differences in the hypoxic tolerance in the CoCl_2_ and NS groups, some molecular induction by hypoxia not regulated by HIF-1 may have contributed to these observed differences [35,36,37]. For example, the inhibitor of apoptosis protein-2 (IAP-2) induced by severe hypoxia and its expression was HIF-1-independent [36]. IAP-2 was resistant to cytochrome c-stimulated caspase activation that caused cell death [38]. There are more than 100 HIF-1 downstream genes identified with varying functions [34,39]. These genes, such as *IAP-2*, which are not regulated directly by HIF-1, could play a marginal role in the differences of hypoxic tolerance in hypoxia and CoCl_2_ treatment we observed; nonetheless, the role of HIF-1 as the key regulator of adaptive responses under hypoxic conditions needs further study

## 3. Experimental Section

### 3.1. Animal Model

A total of 96 male adult Balb/c mice (16.0–22.0 g) were divided into two groups of 48 mice prior to hypoxia exposure; group 1 was treated with CoCl_2_ (injected with 60 mg/kg, [40,41]) and group 2 with normal saline (NS). The procedure of hypoxic experiment was performed as previously described [9]. The mice were placed into a 125 mL jar with fresh air, which was sealed with a rubber plug. The animal was removed from the jar as soon as gasping breath appeared and switched to a second similar fresh air jar that was immediately sealed again. The appearance of the first gasping was regarded as the tolerance limit in each trial. The time period between the beginning of airtightness and the appearance of the first gasping was termed “tolerance time” or “hypoxia tolerance” for each run. This procedure was repeated for four times (H4 group), one time (H1 group) or zero time (H0 group). This study followed the US National Institutes of Health principles for laboratory animal care, and the protocol was approved by the Committee on the Ethics of Animal Experiments (No. 20100608) of the Baotou Medical College.

### 3.2. Western Blot Analysis

Total proteins from mouse hippocampus were extracted according to a previously described protocol [42]. Protein concentrations were determined using bicinchoninic acid (BCA) method. Total cell lysate of mouse hippocampus (80 μg) was separated by 8% SDS-PAGE at 30 mA for 2.5 h and then blotted onto a nitrocellulose membrane. The membrane was then incubated for 1 h in blocking buffer (Tris-buffered saline (TBS) containing 10% skimmed-milk powder) at room temperature. Next, the membrane was incubated for 16 h at 4 °C with the primary antibodies, then incubated with secondary antibodies for 1 h at room temperature. After each incubation the membranes were washed three times thoroughly with TBS. Protein signals were detected by ECL (Pierce Chemical Company, Rockford, IL, USA) detection system in which the membrane was exposed to the detection solution for 5 min.

### 3.3. Electrophoretic Mobility Shift Assay (EMSA)

EMSA was performed according to the method of Semenza and Wang [43]. The *EPO* gene-derived sense oligonucleotide sequence 5'-GCCCTACGTGCTGCCTCGCATGGC-3' contains a HIF-1-bindingsite. Sense and antisense oligonucleotides were annealed in annealing buffer and then were labeled with [γ-p^32^] ATP by T4 polynucleotidekinase (Takara Holding Company Inc., Dalian, China). Unincorporated nucleotides were removed by using the QIAquick nucleotide removal kit (Qiagen Inc., Valencia, CA, USA). Nuclear protein form hippocampus was added to binding buffer which with or without antiboby [7]. Then the mixture was incubated on ice for 30 min before addition of 1 × 10^4^ cpm of oligonucleotideprobe in a total volume of a 20 μL solution for one additional hour. Samples were run on 4% nondenaturing polyacrylamide gels at 200–250 V for 2–3 h in 0.5× TBE buffer (Tris–borate–EDTA) at 4 °C. Afterwards, the gels were dried and auto-radiographed.

### 3.4. Hippocampal Slice Preparation and PS Recording

Hippocampal slices (400-μm thickness) were prepared from adult male mice. After sacrifice, the brain was quickly removed and placed in ice-cold, oxygenated artificial cerebrospinal fluid (ACSF) for about one minute. The hippocampal slices were prepared essentially as previously indicated [9]. Freshly prepared slices were maintained in ACSF at room temperature for at least 3 h before recording.

A single hippocampal slice was then transferred to the recording chamber, in which it was held submerged between two nylon nets and maintained at 32 °C. The mixed gas flushed constantly on the upper surface of slice and the warm ACSF (30 °C) flushed under the lower surface with opposite direction of gas. The hypoxia stimulus was 95% N_2_ and 5% CO_2_. Extracellular recordings population spikes (PSs) were obtained from the slice using microelectrodes filled with 4 M NaCl (resistance 2–10 MΩ).

### 3.5. RNA Isolation, cDNA Synthesis, and Real-Time PCR

Total RNA was prepared from mouse hippocampus using the Rneasy Mini Kit (Qiagen Inc.) and total RNA (1 µg) was reverse-transcribed with Superscrip II reverse transcriptase (Qiagen Inc.). The 1 μL reverse transcription product was used for real-time PCR. Real-time PCR reactions were performed in a volume of 25 μL containing a pair of specific primers and 12.5 μL SYBR green Master Mix (Boerdi, Nanjing, China) using the following conditions: Initial denaturing: 95 °C for 15 min, followed by 40 cycles of denaturing at 95 °C for 30 s, annealing and extension at 60 °C for 65 s. The primer sequences used for real-time PCR are as follows: *EPO*-forward: GGAGACCTTACCACTTGAAGATG; *EPO*-reverse: GCCCGGAACCTATCTATCCTCT; *VEGF*-forward: GTGAGGTGTGTATAGATGTGGGG; *VEGF*-reverse: ACGTCTTGCTGAGGTAACCTG; *â-actin*-forward: GGCTGTATTCCCCTCCATCG; β-*actin*-reverse: CCAGTTGGTAACAATGCCATGT.

The threshold cycle number (*C*_t_) value for *EPO* and *VEGF* were normalized against β-*actin* reference genes and calculated as Δ*C*_t_ = (*C*_t,EPO_ − *C*_t,β-Actin_) or Δ*C*_t_ = (*C*_t,VEGF_ − *C*_t,β-Actin_). Relative *EPO* or *VEGF* mRNA concentrations were expressed as folds versus reference (*F* = 2^Δ*C*t^).

### 3.6. Quantification and Statistical Analysis

The optical density (OD) of bands of Western Blot and EMSA was obtained through the Gel-Doc analysis system and analyzed by Bandscan software (Glyko, Novato, CA, USA). The data of real-time PCR is present as F and Western Blots is presented as relative abundance (RA, and EMSA is presented as OD. Non-parametric Mann-Whitney *U* test and Student’s independent *t*-test were used, with an α of ≤0.05 considered to be statistically significant.

## 4. Conclusions

Taken together, the present study showed that CoCl_2_ pre-treatment increases hypoxic tolerance time, the abolishment time of PS, HIF-1α level, HIF-1 target genes *EPO* and *VEGF* in a sort of plateau that is no longer inducible by hypoxia and repeated acute hypoxic stimuli. At the same time, CoCl_2_ administration attenuated HIF-1 DNA binding activity which can be induced by repeated acute hypoxia. Although the mechanisms are not fully elucidated, there may be other molecular mechanisms, in addition to HIF-1 activity, which are involved in regulation of the expressions of HIF-1 target genes. The findings of this study reveal that HIF-1 is important for hypoxia tolerance. Other mechanisms that can be inhibited by over-induced HIF-1α may be involved in hypoxia tolerance induced by repetitive autohypoxia.

## Supplementary Files

Supplementary File 1 Supplementary Information (PDF, 678 KB)
