# Supplementary material for: The Effects of CoCl2 on HIF-1α Protein under Experimental Conditions of Autoprogressive Hypoxia Using Mouse Models
Source: Int J Mol Sci. 2014 Jun 18;15(6):10999–1012. doi: 10.3390/ijms150610999 (PMC4100194; doi:10.3390/ijms150610999)
Supplement: Supplementary File 1 — Supplementary Information (PDF, 678 KB) [file ijms-15-10999-s001.pdf]

Supplementary Information

Figure S1. Expression of HIF-1α and β-actin at different time point after $CoCl_2$ treatment.

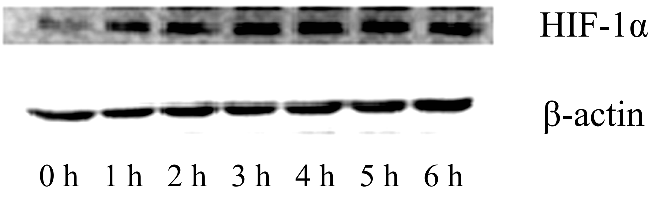

Figure S2. Ratio of HIF-1α protein to β-actin protein at different time point.

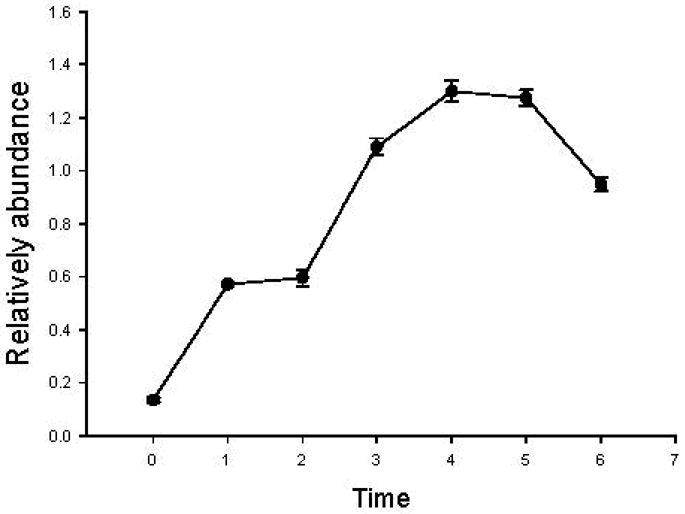

© 2014 by the authors; licensee MDPI, Basel, Switzerland. This article is an open access article distributed under the terms and conditions of the Creative Commons Attribution license (http://creativecommons.org/licenses/by/3.0/).
